# A deep learning dataset for metal multiaxial fatigue life prediction

**DOI:** 10.1038/s41597-024-03862-4

**Published:** 2024-09-19

**Authors:** Shuonan Chen, Yongtao Bai, Xuhong Zhou, Ao Yang

**Affiliations:** 1 School of Civil Engineering, Chongqing University, Chongqing, 400045 China; 2 Research Center of Steel Structure Engineering, Chongqing University, Chongqing, 400045 China

**Keywords:** Mechanical engineering, Metals and alloys

## Abstract

Multiaxial fatigue failure of metals, a common issue in industrial production, often leads to significant losses. Recently, many researchers have applied deep learning methods to predict the multiaxial fatigue life of metals, achieving promising results. Due to the high costs of fatigue testing, training data for deep learning is scarce and labor-intensive to collect. This study meets this need by creating a large-scale, high-quality dataset for multiaxial fatigue life prediction, consisting of 1167 samples from 40 materials collected from literature. The dataset includes key mechanical properties (elastic modulus, yield strength, tensile strength, Poisson’s ratio) and 48 loading paths, along with additional relevant information (composition ratios, processing conditions). Common deep learning models validated the dataset’s effectiveness. This dataset aims to support researchers applying deep learning to fatigue life prediction, addressing the long-standing issue of data scarcity, thereby advancing the intersection of artificial intelligence and metal fatigue research.

## Background & Summary

Metal fatigue, a critical phenomenon affecting the integrity and lifespan of structural components, poses significant challenges across various engineering domains^1^. One of the key points of interest in fatigue analysis is the prediction of fatigue life. Traditional fatigue analysis methods, such as strain energy-based^2,3^ and critical plane approaches^4–7^, while effective, often require extensive experimental data and are constrained by their specificity to particular materials and loading conditions^8,9^. The advent of advanced computational techniques, particularly deep learning, offers a promising avenue to overcome these limitations by enabling the prediction of fatigue life across diverse scenarios.

In recent years, methods such as Deep Neural Networks (DNN), Long Short-Term Memory networks (LSTM), Convolutional Neural Networks (CNN), Physics-Informed Neural Networks (PINN), Bayesian Neural Networks, and self-attention mechanisms have been successfully applied to the prediction of fatigue life in various materials and structures^10–19^. Results indicate that these approaches can effectively identify complex multiaxial loading paths, demonstrating excellent performance. These models have shown potential in fields ranging from image recognition to natural language processing, and their application in materials science is increasingly gaining traction^20^. Currently, there is a lack of publicly available high-quality deep learning datasets for metal fatigue. One of the major characteristics of deep learning is its high data dependency^21^, which undoubtedly hinders the development of applying deep learning techniques in fatigue analysis.

Therefore, we aim to address this gap by constructing a comprehensive and high-quality dataset that encapsulates the multifaceted nature of metal fatigue under various loading conditions. This dataset encompasses a broad spectrum of metals, including but not limited to, aluminum alloys, steel alloys, and titanium alloys, each tested under various cyclic loading conditions such as uniaxial tension-compression, multiaxial loading, and non-proportional loading paths. The dataset is enriched with detailed descriptions of material properties, loading parameters, and resulting fatigue lives, providing a robust foundation for developing predictive models. The significance of this dataset lies in its potential to facilitate the development of generalized deep learning models capable of accurately predicting metal fatigue life across different materials and loading conditions. By making this dataset publicly available, we invite researchers in the field of metal fatigue to use this comprehensive resource as a foundation for advancing their work. Leveraging the extensive data we have curated, researchers can develop and refine predictive models, perform detailed analyses, and explore new hypotheses with greater accuracy and efficiency.

Moreover, we encourage the research community to contribute to and enhance this dataset by sharing additional data points, experimental results, and insights. Collaborative efforts will help to continually improve the quality and breadth of the dataset, making it an increasingly powerful tool for fatigue analysis. This contribution not only underscores the transformative potential of deep learning in fatigue analysis but also sets a benchmark for future datasets in the field. The comprehensive nature of the dataset ensures that it can serve as a valuable resource for both the validation of existing models and the exploration of novel deep learning architectures tailored to fatigue life prediction.

## Methods

### Data collection

To develop a comprehensive dataset for predicting metal fatigue life using deep learning, we conducted an extensive literature review using Google Scholar and Web of Science, using the search keyword “metal multiaxial fatigue”. Our search strategy focused on identifying experimental studies that reported fatigue data for various metals under different loading conditions. The selection criteria were designed to encompass a wide range of materials and loading paths to ensure the dataset’s diversity and applicability.

### Materials

In the selected papers, detailed information about the specimens is typically provided in sections such as “Materials” or “Experiment”. This information includes the specimen dimensions, mechanical properties, chemical composition, and processing methods. We manually extracted and organized these details to ensure comprehensive coverage of each material’s characteristics. Key mechanical properties such as elastic modulus, tensile strength, yield strength, and Poisson’s ratio were tabulated.

### Experiment

The experimental setup and procedures are generally described in sections like “Fatigue Test”. We focused on extracting critical details about the loading conditions, including the waveform used, stress amplitude, and stress ratio. These parameters are essential for understanding the loading path and subsequent vectorization for deep learning applications. All data were derived from standardized multiaxial fatigue tests, with temperature kept constant at room temperature, except for GH4169 and Hayes, which were tested under high-temperature conditions. For each sample, we followed consistent processing steps to ensure that all procedures across different experiments were identical. This consistency ensures the reliability and comparability of our data.

**Fig. 1:**
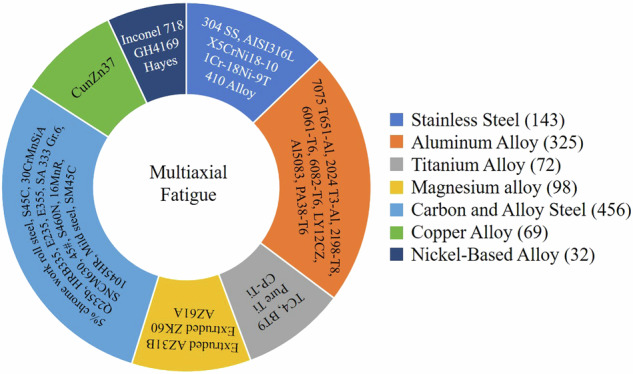
Schematic diagram of the types of materials collected in the dataset. The dataset comprises six categories of metallic materials, each including detailed loading paths and mechanical properties for every sample.

Among the over 70 literature sources reviewed, many did not provide the necessary mechanical properties or loading path information. Such data points were not included in our dataset. Ultimately, we retained data from 36 papers, encompassing 40 different metallic materials, including 7 types of materials including stainless steel, aluminum alloy, titanium alloy, magnesium alloy, alloy steel, copper alloy, and nickel alloy, resulting in a total of 1,167 data points, as illustrated in Fig. 1. The data points are divided into two groups: one consists of stress-controlled experimental data points, and the other consists of strain-controlled experimental data points. The Poisson’s ratio of some of the materials was not directly given and was calculated according to Eq. 1. In the equation, *G* (Pa) represents the shear modulus, *E* (Pa) is the elastic modulus, and *ν* represents the Poisson’s ratio. For specimens subjected to the same loading conditions and tested multiple times, the fatigue life in this dataset represents the average value. It should be noted that Eq. 1 is specifically valid for isotropic and homogeneous materials, which are implicitly expected to be the types of materials included in the database.

1 $$G=E/2(1+v)$$

These data points span 48 distinct loading paths, capturing the complex interactions between material properties and fatigue behavior. The primary sources of these data were experimental studies that presented their findings in tabular formats, typically detailing the material properties, loading conditions, and observed fatigue lives.

### Data processing

**Fig. 2:**
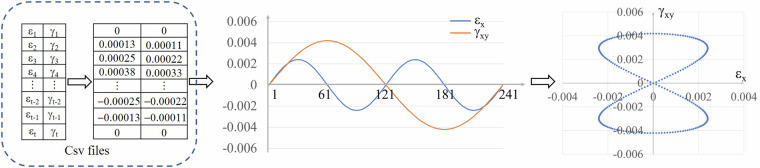
Loading paths are saved in CSV files. The vectorized loading paths information is stored in CSV files, with graphical representations generated from two columns of data accurately capturing the essential characteristics of the loading paths, including amplitude, shape, mean stress, etc.

**Fig. 3:**
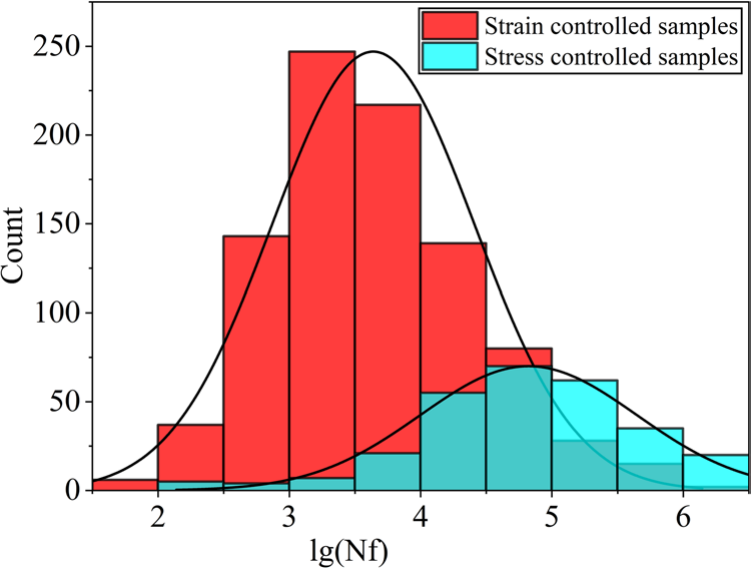
Schematic diagram of the types of materials collected in the dataset. The dataset comprises six categories of metallic materials, each including detailed loading paths and mechanical properties for every sample.

The mainstream methods currently applied in fatigue life prediction generally involve predicting the fatigue life of materials based on their mechanical properties and loading paths^11,12,18^. In real-world applications, fatigue loads are often highly complex, involving variations in amplitude, mean stress, and loading frequency. Vectorizing the loading path allows us to comprehensively capture these critical aspects and represent them as temporal sequences for neural network input. This approach enables neural networks to capture the sequential dependencies and complex patterns within the loading cycles, which is crucial for accurate fatigue life prediction^11,18^. Furthermore, vectorizing the loading path as temporal information enables neural networks to capture the sequential dependencies and complex patterns within the loading cycles, which is essential for accurate fatigue life prediction. Therefore, we manually vectorized 1,167 samples from 40 different materials, setting the sequence length t to 241. The loading information for each sample is stored in a separate CSV file. As shown in Fig. 2, the first column in the CSV file represents axial stress σ_t_ or strain ε_t_, while the second column represents shear stress τ_t_ or strain *γ*_t_. The CSV filenames correspond to the actual specimens documented in the literature, with the filenames and corresponding mechanical properties listed in another CSV file for consolidation, which we will refer to as the summary CSV file. As illustrated in Table 1, the first column on the left lists the filenames of the loading path CSV files, and columns two to five correspond to each specimen’s elastic modulus, tensile strength, yield strength, and Poisson’s ratio, respectively. The final column represents the logarithm of the corresponding fatigue life, denoted as lg(Nf). The distribution of fatigue life of the entire dataset samples is shown in Fig. 3.

**Table 1:** Dataset overview.

Loading paths	Young’s modulus: E (Gpa)	Ultimate tensile strength: σ_u_ (Mpa)	Yield strength: σ_y_ (Mpa)	Poisson’s ratio: ν	The logarithm of fatigue life: lg(N_f_)
q235b-0.002.CSV	206	412	235	0.30400	4.427583473
q235b-0.003.CSV	206	412	235	0.30400	4.427583473
⋮	⋮	⋮	⋮	⋮	⋮
SNCM630-TA05.CSV	196	1103	951	0.27300	3.287129621

The first column on the left is the CSV file name of the loading path of the specimen. The second to fifth columns correspond to the elastic modulus (E), tensile strength (σ_u_), yield strength (σ_y_), and Poisson’s ratio (*v*) of each specimen, respectively. The last column is the logarithm lg(Nf) of the corresponding fatigue life.

The unbalanced nature of a dataset is a common issue in predictive modeling. Common strategies to address unbalanced datasets include resampling techniques such as oversampling and undersampling, data augmentation, and adjusting the cost function to more severely penalize misclassifications of minority classes. The fact that our target data approximately follows a normal distribution indicates that the distribution of our data is relatively balanced, as shown in Fig. 3. In this case, additional measures to address data imbalance are generally not required. However, we remain vigilant and are prepared to implement these strategies should any imbalance issues arise in future datasets or different applications.

Since stress-controlled loading typically corresponds to values in the range of hundreds of MPa, while strain-controlled loading values are often only a few thousandths, their vectorized forms have inconsistent units. Although there is a conversion relationship between stress and strain, to ensure the accuracy and authenticity of the data, we did not apply such conversions. Instead, we distinguished between the two, storing the corresponding CSV files in separate folders and creating respective summmarized CSV files (Table 1).

**Fig. 4:**
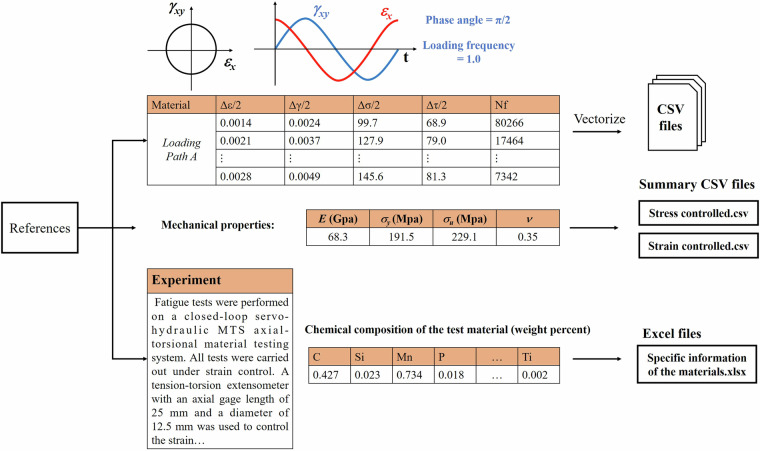
Distribution of the collected samples. The red part represents samples under strain-controlled tests, while the blue part represents samples under stress-controlled tests. The distribution of fatigue life in both parts of the samples is roughly similar to a normal distribution.

The entire data collection process is illustrated in the Fig. 4, providing a reference for researchers interested in expanding this dataset. By continuously enriching the dataset, this initiative aims to advance the application of deep learning in the field of multiaxial fatigue life prediction.

Figure 4 Flow chart of data collection process. After reviewing the relevant literature, the loading path information was vectorized and stored in CSV files. Each material subjected to multiaxial fatigue testing has multiple loading paths, which may vary in stress amplitude, stress ratio, and frequency ratio. The mechanical properties of each test material are typically found in the Materials section of the literature and can be organized into a Summary CSV file. Additional experimental details are generally available in the Experiment section and can be compiled into a Specific Information of the Materials.xlsx file.

## Data Records

The entire dataset^22^, including the individual CSV files and the summary CSV spreadsheet (Table 1), has been made publicly available on Materials Cloud at 10.24435/materialscloud:ad-xk. “Specific information of the materials” provides detailed information about the collected materials. The specific loading path of each sample is stored as time series data in CSV files. These CSV files are organized into two folders named “data_all_strain_controlled” and “data_all_stress_controlled”, which respectively record the loading paths of strain-controlled and stress-controlled specimens. The two similarly named CSV files summarize the mechanical properties of each specimen corresponding to their respective loading paths, as shown in Table 1.

**Fig. 5:**
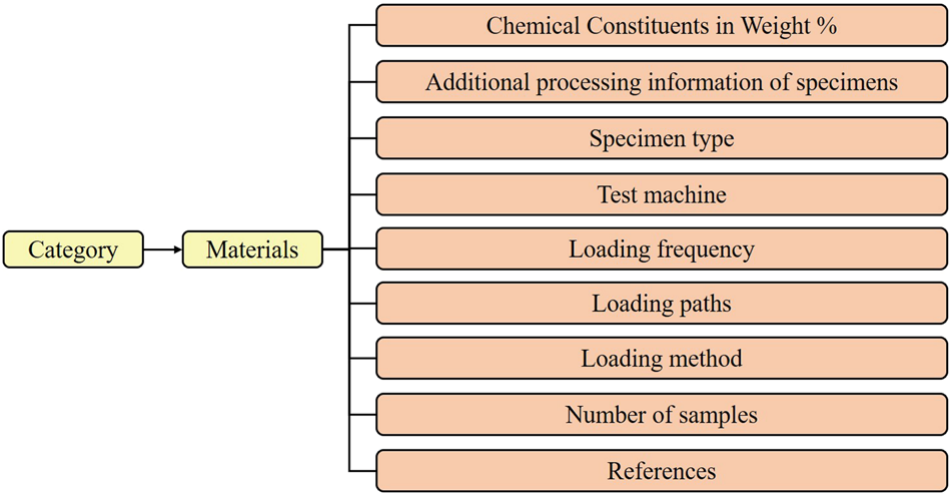
The information index structure for each material. Users can easily access any other necessary information by referring to this index. These details are stored in an Excel file named “Specific information of the materials”.

The additional Excel file serves as an index for users to access supplementary information. Based on the dataset of high-entropy alloys designed in previous referenced literature^23^, this index includes detailed information on each material and additional metadata. The specific information of this index is shown in Fig. 5. By providing this level of detail, we aim to enhance the transparency and reproducibility of our dataset, allowing researchers to trace the origins of the data and understand the context of the experiments. It should be noted that this Excel file is provided solely for the convenience of users to understand the material information and serve as an index, rather than being used for deep learning training tasks.

Researchers can access this dataset^22^ to develop and validate their predictive models, perform meta-analyses, or explore new hypotheses in the field of metal fatigue. The dataset’s structure ensures that it is both comprehensive and user-friendly, promoting widespread adoption and collaborative research efforts.

## Technical Validation

To ensure the accuracy of the data, we manually performed multiple checks and corrections. For the loading path information of each sample, we plotted the two columns of data from each CSV file (the first column represents axial stress or strain, and the second column represents tangential stress or strain, with the first column data as the x-coordinate and the second column data as the y-coordinate). We verified the shape and amplitude of the paths against the original experimental data. During this verification process, we compiled all the loading path shapes involved in the current dataset and presented them in Table 2 for reference.

**Table 2:** Loading paths collected in this dataset.

Loading paths	Diagrams
Uniaxial:	
Proportional:	
Nonproportional I:	
Nonproportional II:	
Nonproportional III:	
Nonproportional IV:	
Nonproportional V:	
Nonproportional VI:	

By controlling the waveform, frequency, and amplitude of the loading, these different loading paths can be obtained.

To ensure the technical quality and applicability of our dataset, we employed several common deep learning algorithms, including GRU (Gate Recurrent Unit), LSTM (Long Short-Term Memory Networks), and 1D CNN (Convolutional Neural Network), integrated with fully connected layers (FCL) to validate the dataset. 1D CNNs are efficient at recognizing local patterns, LSTMs excel at capturing long-term dependencies, and GRUs offer simplified architectures with fewer parameters while still maintaining performance. These deep learning models were chosen for their distinct capabilities in processing and learning from complex, high-dimensional data, which is characteristic of fatigue life prediction tasks. The comparative analysis of these networks provides insights into their respective strengths and suitability for developing robust predictive models.

The models were trained and evaluated using standard metrics such as Mean Absolute Error (MAE), Mean Squared Error (MSE), and R-squared (R²) to ensure their effectiveness in predicting fatigue life. This validation aimed to demonstrate the dataset’s utility in training predictive models for metal fatigue life under various loading conditions.

We trained and tested the aforementioned deep learning models using our dataset. The primary objectives were to:

- Verify that the dataset contains sufficient information to train effective predictive models.
- Ensure that the data structure and format are compatible with standard machine learning workflows.
- Evaluate the accuracy and reliability of the dataset across different materials and loading paths.

**Fig. 6:**
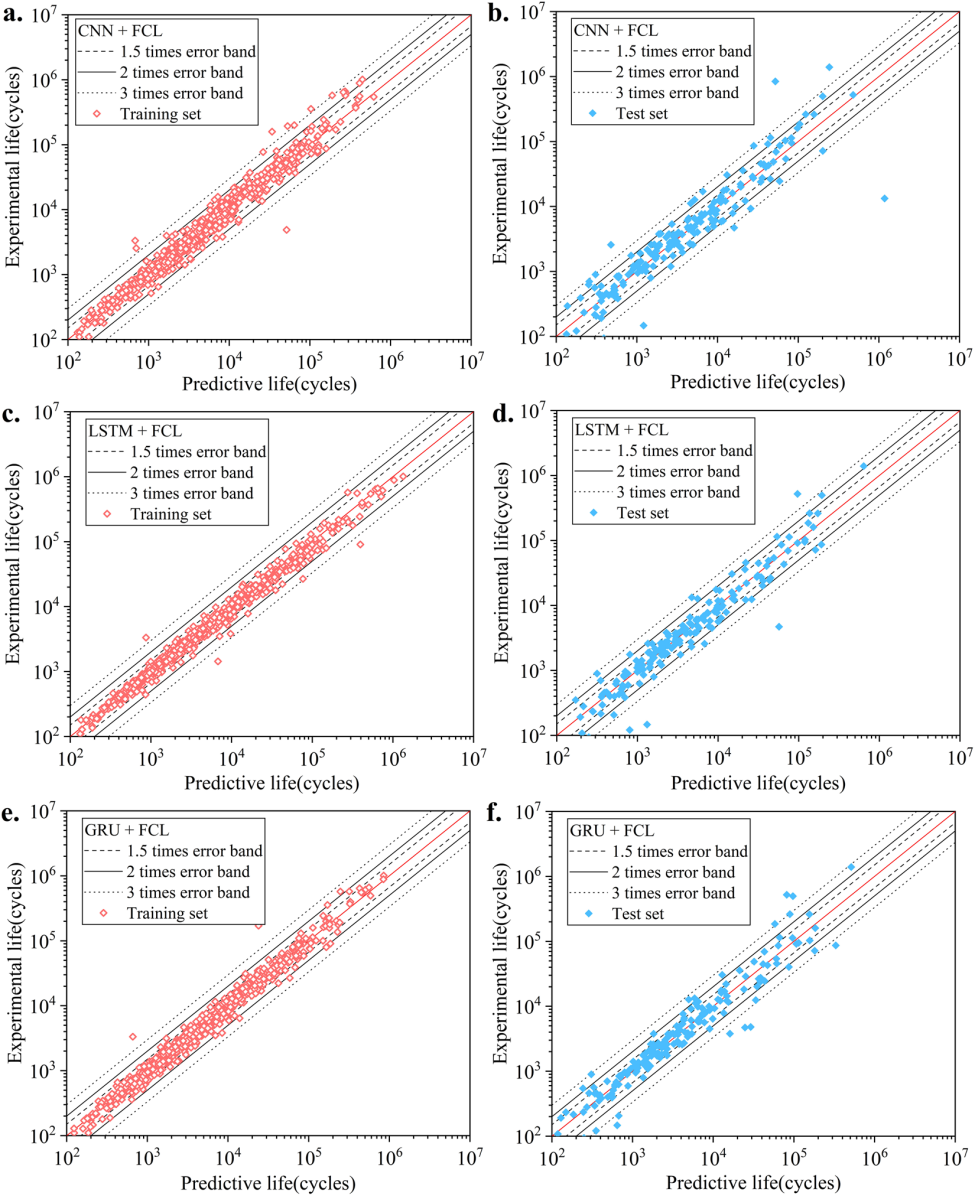
Predicted results under different deep learning methods. We employed relatively basic network architectures with shallow layers and simple parameters. Despite this simplicity, the three types of models exhibited commendable predictive performance, with the majority of the data points falling within three times scatter band. Among these models, the CNN showed relatively poorer performance, whereas the LSTM and GRU models demonstrated better accuracy.

The predicted results are shown in Table 3, and Fig. 6. The models achieved satisfactory performance metrics, indicating that the dataset is of high quality and suitable for developing predictive models. The detailed results are as follows:

**Table 3:** Performance for 3 tested basic deep learning models.

Used model	MAE	MSE	R^2^
CNN + FCL	0.181932	0.116249	0.826342
LSTM + FCL	0.146262	0.059000	0.911863
GRU + FCL	0.155936	0.056612	0.915431

The three metrics indicate that the CNN+FCL architecture performs relatively poorly, with a significantly lower R^2^ and higher MAE and MSE. The other two architectures (LSTM+FCL and GRU+FCL) perform better overall and are able to predict fatigue life more accurately, demonstrating the dataset’s suitability for developing effective predictive models. This suggests that LSTM and GRU are more effective at capturing the critical features of the loading paths provided in the dataset. However, it should be noted that despite the better performance of LSTM and GRU, overfitting may still occur in practical applications, and further model optimization is necessary.

Upon examining the data dispersion, we observed a slight overfitting phenomenon. This is indicated by the performance during the training phase, where the training error is significantly lower than the validation error. However, the overall performance of the models, particularly the LSTM and GRU, still proves to be robust, suggesting that even with basic architectures, deep learning techniques can effectively predict metal fatigue life under various loading conditions.

These results confirm that our dataset meets the necessary standards for technical quality and can serve as a reliable foundation for further research in metal fatigue life prediction. To support the research community, we also provided detailed documentation on data integrity checks and preprocessing steps. We encourage researchers to leverage and contribute to this dataset, facilitating further advancements in the field of metal fatigue analysis using deep learning techniques.

**Fig. 7:**
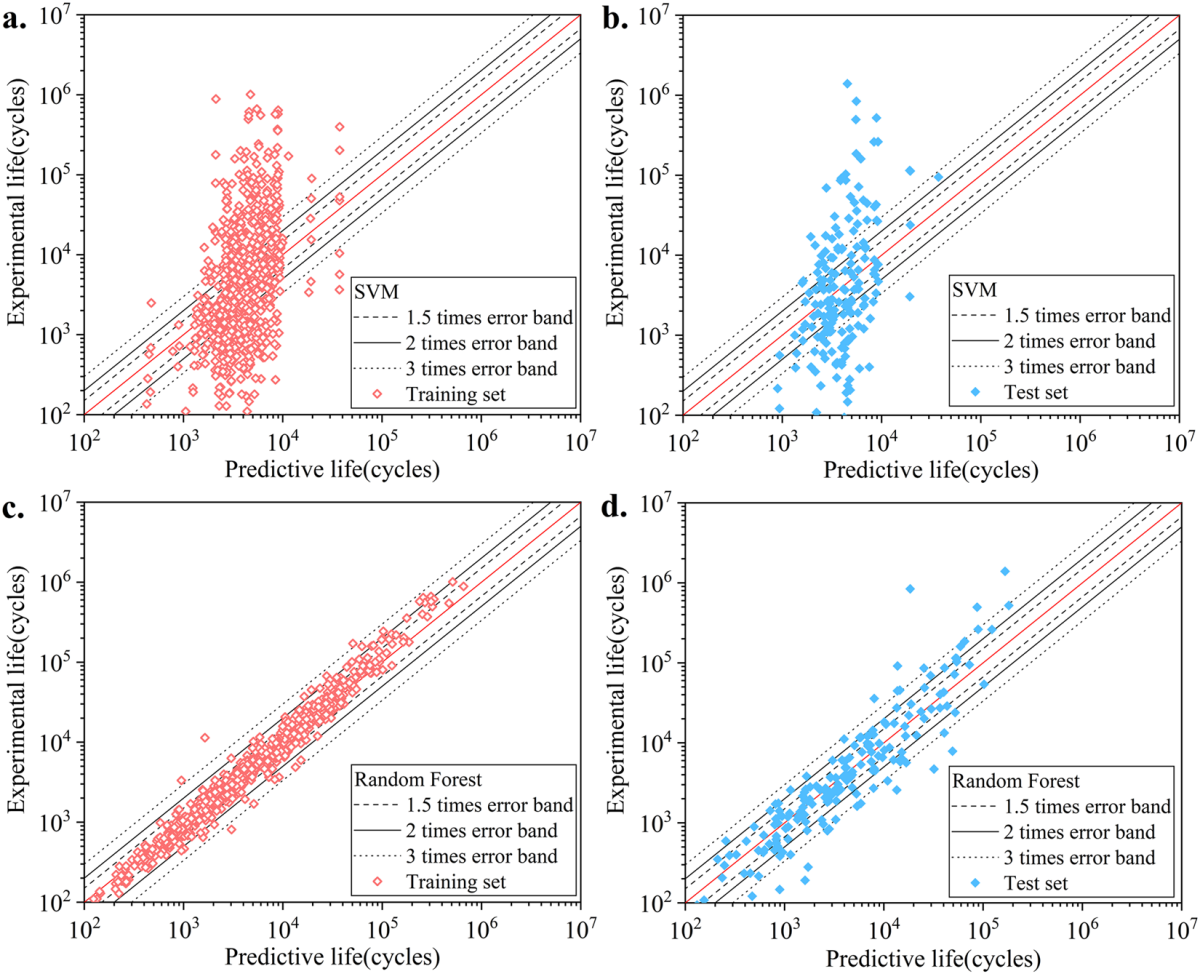
Predicted results under SVM and RF. The results show that the data points predicted by the SVM are highly dispersed, indicating that the SVM failed to capture the relationship between the fatigue loading paths and material properties. RF performs well on the training set, while the test set is relatively more dispersed, but most data points still fall within three times scatter band.

Although this study primarily focuses on deep learning models, we also evaluated traditional machine learning algorithms such as SVM (Support Vector Machine) and RF (Random Forest). The results showed that these classical methods performed worse compared to deep learning architectures. Specifically, SVM performed very poorly, while RF performed relatively better but still fell short of the deep learning models. These findings, as shown in Table 4 and Fig. 7, reinforce the necessity of using advanced neural networks for accurate fatigue life prediction.

**Table 4:** Performance for SVM and RF.

Used model	MAE	MSE	R^2^
SVM	0.553325	0.579562	0.173420
Random Forest	0.147487	0.254978	0.779677

The R² of the SVM is only around 0.17, with both MAE and MSE being quite high, indicating poor predictive performance. On the other hand, the R² of the RF can reach a relatively high value of 0.78, and both MAE and MSE remain at low levels, which is relatively good but still lower compared to the deep learning models.

This result may be attributed to the limitations of its linear or simple kernel function, which is less effective in managing high-dimensional and complex data. The data for fatigue life prediction likely exhibit highly nonlinear and complex feature patterns that SVM fails to effectively capture. Random Forest (RF) performed relatively better because RF is an ensemble learning method that improves prediction accuracy and robustness by combining multiple decision trees. RF can handle nonlinear relationships and capture complex patterns in the data to some extent. However, RF is still limited by the depth and number of its trees and may not be as effective as deep learning models in capturing intricate dependencies and long-term memory features in the data.

## Usage Notes

To enable easy access to our dataset and to support the replication of our results, we have uploaded code examples for the three employed models (CNN, LSTM, and GRU) to GitHub. Researchers can refer to these examples to understand the implementation details and adapt the models for their own studies.

For the mechanical properties, excluding the loading path, it is recommended to perform normalization before splitting the data into training and testing sets. This normalization step ensures that the features are on a similar scale, which is crucial for the effective training of deep learning models. The code provided in our GitHub repository includes this preprocessing step.

**Fig. 8:**
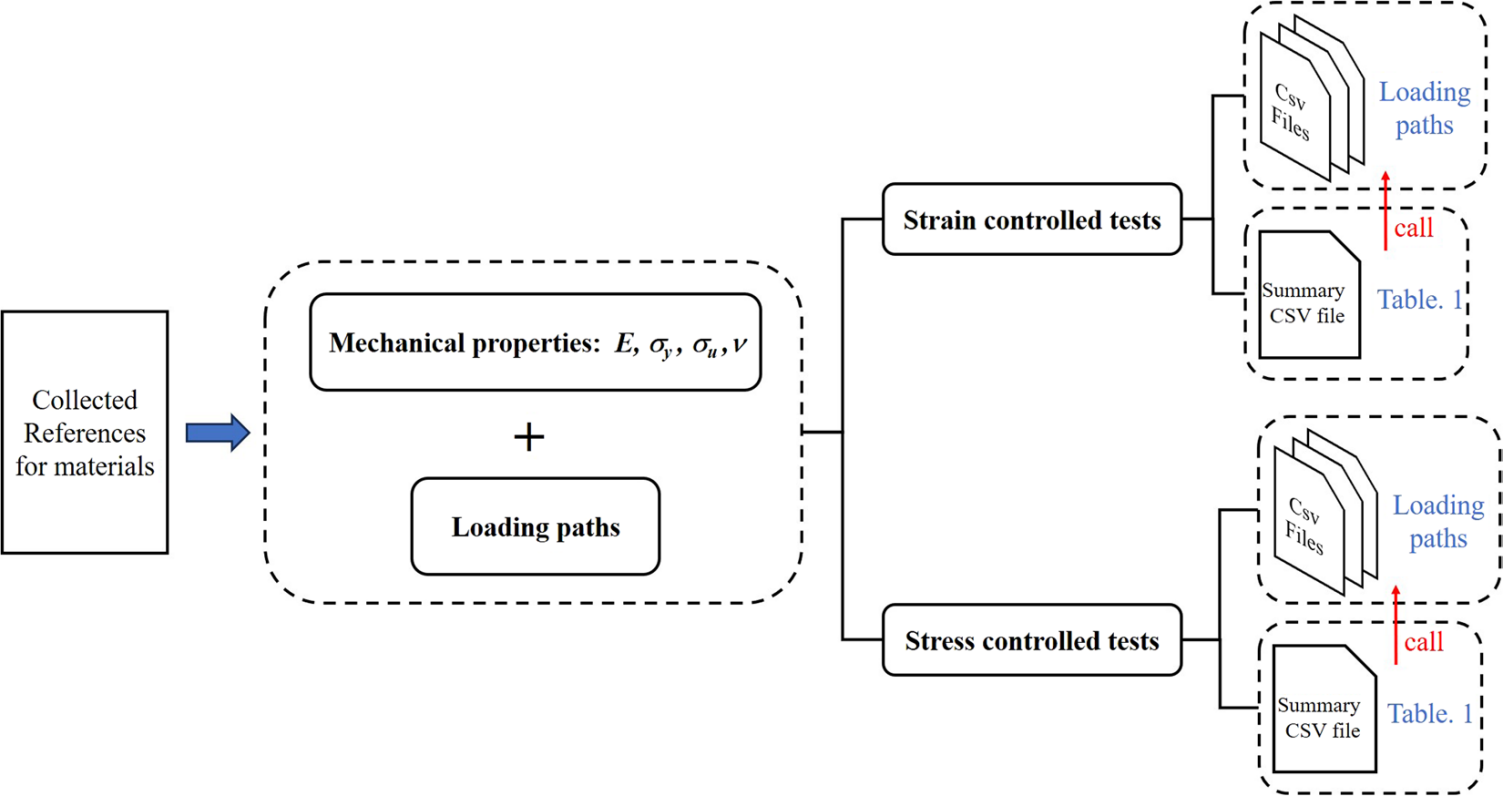
Diagram of the workflow and the file call relationship. As described in Table 1 above, during the deep learning training process, each CSV file containing loading path information can be referenced using the CSV file names listed in the first column of the summary CSV file.

The specific workflow is illustrated in Fig. 8. Researchers can determine how to utilize these files based on their specific study requirements. If the goal is to use deep learning algorithms to study the effect of loading paths on the fatigue life of a specific material, they may choose to only reference the CSV file names listed in the first column of the summary CSV file, without using the other mechanical properties columns as feature inputs. However, if the researchers wish to study the impact of both the loading paths and the mechanical properties on fatigue life, they can use the first five columns as feature inputs adopting an integrated modeling approach (Integrating different types of neural networks to process different types of data). Additionally, if researchers wish to investigate other influencing factors, they can add columns to the dataset as features of interest. We hope that the proposed dataset can serve as a valuable starting point for further research.

Researchers interested in considering additional features can extend the dataset by adding more columns to the summary CSV file (Table 1) following the provided format. This flexibility allows for the incorporation of various mechanical properties and other relevant features that might influence fatigue life prediction.

## Data Availability

The code for validating the dataset using the deep learning algorithms is freely available on GitHub: https://github.com/stupid-cooh/Metal-Multiaxial-Fatigue-Life-Prediction-Using-Deep-Learning/tree/main. Usage instructions and specific details can be found in the code docum-entation.

## References

[1] Stephens, R. I., Fatemi, A., Stephens, R. R. & Fuchs, H. O. *Metal Fatigue in Engineering*. (John Wiley & Sons, 2000).

[2] Brown, M. W. & Miller, K. J. A Theory for Fatigue Failure under Multiaxial Stress-Strain Conditions. *Proc. Inst. Mech. Eng.* **187**, 745–755 (1973).

[3] Wang, C. H. & Brown, M. W. A Path‐Independent Parameter for Fatigue Under Proportional and Non‐Proportional Loading. *Fatigue Fract. Eng. Mater. Struct.* **16**, 1285–1297 (1993).

[4] Fatemi, A. & Socie, D. F. A Critical Plane Approach to Multiaxial Fatigue Damage Including Out-of-Phase Loading. *Fatigue Fract. Eng. Mater. Struct.* **11**, 149–165 (1988).

[5] Smith, K. A stress-strain function for the fatigue of metals. *J. Mater.* **5**, 767–778 (1970).

[6] Liu, K. C. A method based on virtual strain-energy parameters for multiaxial fatigue life prediction. in *Advances in multiaxial fatigue* (ASTM International, 1993).

[7] Chu, C.-C. Fatigue damage calculation using the critical plane approach. (1995).

[8] Cui, W. A state-of-the-art review on fatigue life prediction methods for metal structures. *J. Mar. Sci. Technol.* **7**, 43–56 (2002).

[9] Kamal, M. & Rahman, M. M. Advances in fatigue life modeling: A review. *Renew. Sustain. Energy Rev.* **82**, 940–949 (2018).

[10] Gao, J., Heng, F., Yuan, Y. & Liu, Y. A novel machine learning method for multiaxial fatigue life prediction: Improved adaptive neuro-fuzzy inference system. *Int. J. Fatigue* **178**, 108007 (2024).

[11] Yang, J., Kang, G., Liu, Y. & Kan, Q. A novel method of multiaxial fatigue life prediction based on deep learning. *Int. J. Fatigue* **151**, 106356 (2021).

[12] Yang, J., Kang, G. & Kan, Q. A novel deep learning approach of multiaxial fatigue life-prediction with a self-attention mechanism characterizing the effects of loading history and varying temperature. *Int. J. Fatigue* **162**, 106851 (2022).

[13] Zhang, X.-C., Gong, J.-G. & Xuan, F.-Z. A deep learning based life prediction method for components under creep, fatigue and creep-fatigue conditions. *Int. J. Fatigue* **148**, 106236 (2021).

[14] Sun, X., Zhou, T., Song, K. & Chen, X. An image recognition based multiaxial low-cycle fatigue life prediction method with CNN model. *Int. J. Fatigue* **167**, 107324 (2023).

[15] Jia, Y. *et al*. Fatigue life prediction based on a deep learning method for Ti-6Al-4V fabricated by laser powder bed fusion up to very-high-cycle fatigue regime. *Int. J. Fatigue* **172**, 107645 (2023).

[16] Jarrah, M. A., Al-Assaf, Y. & Kadi, H. E. Neuro-Fuzzy Modeling of Fatigue Life Prediction of Unidirectional Glass Fiber/Epoxy Composite Laminates. *J. Compos. Mater.* **36**, 685–700 (2002).

[17] Chen, D., Li, Y., Liu, K. & Li, Y. A physics-informed neural network approach to fatigue life prediction using small quantity of samples. *Int. J. Fatigue* **166**, 107270 (2023).

[18] Heng, F. *et al*. Multiaxial fatigue life prediction for various metallic materials based on the hybrid CNN-LSTM neural network. *Fatigue Fract. Eng. Mater. Struct.* **46**, 1979–1996 (2023).

[19] Gulgec, N. S., Takáč, M. & Pakzad, S. N. Structural sensing with deep learning: Strain estimation from acceleration data for fatigue assessment. *Comput.-Aided Civ. Infrastruct. Eng.* **35**, 1349–1364 (2020).

[20] Chen, J. & Liu, Y. Fatigue modeling using neural networks: A comprehensive review. *Fatigue Fract. Eng. Mater. Struct.* **45**, 945–979 (2022).

[21] Goodfellow, I., Bengio, Y. & Courville, A. *Deep Learning*. (MIT Press, 2016).

[22] Chen, S. *et al*. A deep learning dataset for metal multiaxial fatigue life prediction. *Materials Cloud Archive*10.24435/materialscloud:ad-xk (2024). 10.1038/s41597-024-03862-4 PMC11413193 39300134

[23] Chen, S. *et al*. Fatigue dataset of high-entropy alloys. *Sci. Data* **9**, 381 (2022). 35794115 10.1038/s41597-022-01368-5 PMC9259632

